# Management of patients with chronic rhinosinusitis with nasal polyps in Spain: learnings from a nationwide survey of otorhinolaryngologists

**DOI:** 10.1007/s00405-023-08185-5

**Published:** 2023-09-01

**Authors:** Isam Alobid, Rafael Fernández Liesa, Jose Miguel Villacampa Aubá, Abraham L. Moure, M. Guadalupe Sánchez-Herrero, Alfonso del Cuvillo Bernal

**Affiliations:** 1 Rhinology and Skull Base Unit, ENT Department, Hospital Clinic, University of Barcelona, Barcelona, Spain; 2 Rhinology and Anterior Skull Base Unit, ENT Department, Hospital Universitario Miguel Servet, Zaragoza, Spain; 3 ENT and Cervicofacial Surgery Department, Fundación Jiménez Díaz, University Hospital, Madrid, Spain; 4 Specialty Care Medical Department, GSK, Madrid, Spain; 5 Rhinology and Asthma Unit, ENT Department, University Hospital of Jerez, Jerez de La Frontera, Cadiz, Spain

**Keywords:** Nose diseases, Nasal polyps, Inflammation, Biological therapy, Surveys and Questionnaires, Patient reported outcome measures

## Abstract

**Purpose:**

To describe the self-reported practices on the diagnosis, treatment, and follow-up of patients with chronic rhinosinusitis with nasal polyps (CRSwNP) by ear, nose, and throat (ENT) specialists in Spain to identify potential areas for management optimization.

**Methods:**

A cross-sectional online survey with 16 questions was carried out. Recruitment was performed by emailing registered ENT specialists in the Spanish Society of Otorhinolaryngology and Head and Neck Surgery (SEORL-CCC).

**Results:**

In total, 127 ENT specialists completed the survey. Fifty-one percent of respondents combined clinical criteria and objective evidence of mucosal inflammation to diagnose CRSwNP. Patient interview and, to a lower degree, a visual analogue scale were the most employed diagnostic tools to quantify symptom severity. Less than half (45%) routinely used the 22-item sino-nasal outcomes test (SNOT-22) to assess the impact of CRSwNP disease in quality of life. The use of patient-reported outcomes and other clinical evaluation tools showed a larger uptake among ENT specialists that worked at an ENT department with an available rhinology unit. Almost all the specialists surveyed (95%) recommended biological treatment, particularly in patients with uncontrolled CRSwNP with respiratory comorbidities (76%), as well as in candidates for revision surgery (66%).

**Conclusion:**

Spanish otorhinolaryngologists showed a trend toward incorporating CRSwNP guideline recommendations in their clinical practice. The observed low uptake of patient-reported outcomes and objective clinical evaluation tools in routine clinical practise have been identified as areas for optimizing the management of patients with CRSwNP.

**Supplementary Information:**

The online version contains supplementary material available at 10.1007/s00405-023-08185-5.

## Introduction

Chronic rhinosinusitis with nasal polyps (CRSwNP) is a chronic inflammatory disease of the mucosa in the nasal and paranasal sinuses, causing symptoms lasting 12 weeks or longer characterized by the presence of bilateral nasal polyps [1, 2]. CRSwNP can significantly impact a patient’s quality of life (QoL) [3], resulting in a substantial socioeconomic burden in terms of productivity loss and healthcare consumption [4, 5], particularly in those with severe and/or uncontrolled disease [2]. Although the multifactorial pathogenesis of CRSwNP is not fully understood, it has traditionally been associated with type 2 chronic inflammation in Western countries, involving local immunoglobulin E (IgE) synthesis, cytokine overproduction and eosinophilic infiltration [6], along with the overexpression of eosinophil cationic protein and formation of Charcot-Leyden crystals [7, 8].

According to international guidelines, once the patient has been carefully evaluated and their phenotype defined, the standard of care for type-2 CRSwNP includes intranasal corticosteroids and saline irrigation as first-line treatment, and short courses of systemic corticosteroids and endoscopic sinus surgery (ESS) as an option for patients with severe, uncontrolled disease [1, 2]. However, a large proportion of severe patients with severe CRSwNP are refractory to the recommended medical-surgical therapy approach and may require add-on treatment with recently approved biological therapies that act on specific targets of the pathogenesis of chronic mucosal inflammation.

Given the variety of treatment options, the highly variable inflammation pattern, and the severity of the disease, patients are usually managed based on a combination of clinical guidelines and expert clinical judgment, as highlighted by previous studies [9–12]. Indeed, treatment approaches may vary among different geographical areas and medical specialties [13], and routine clinical practice may be heterogeneous in terms of diagnosis, phenotyping, treatment schedules, and patient follow-up patterns. To evaluate these aspects, the Rhinology Committee of the Spanish Society of Otorhinolaryngology and Head and Neck Surgery (SEORL-CCC) developed and distributed a nationwide online survey among its members. The objectives were to describe current trends regarding self-reported practices and perceptions on the management of patients with CRSwNP, evaluate compliance with recommendations stated in international guidelines, and identify potential areas for management optimization.

## Methods

### Study design and eligible participants

A cross-sectional, nationwide, online questionnaire-based study was carried out from 21 March to 17 May 2022, among ENT specialists managing patients with CRSwNP across different regions in Spain. Eligible participants were ENT specialists who were members of the SEORL-CCC and willing to participate in this study.

### Study questionnaire and data collection

The SEORL-CCC Rhinology Committee composed by rhinology experts IA, RFL, JMVA, and ACB reviewed current CRSwNP guidelines and designed a questionnaire of 16 items to evaluate the clinical practice and perceptions of ENT specialists on the diagnosis, treatment, and follow-up of patients with CRSwNP in Spain. The questionnaire was divided into three domains: (a) assessment tools for CRSwNP (items 1–6); (b) medical and surgical management of CRSwNP (items 7–12); and (c) patient follow-up visits (items 13–16) [Online resource Appendix 1].

The survey was developed and implemented using an electronic platform (SurveyMonkey®; Momentive Inc., San Mateo, CA, US), which was used as the data collection instrument. An invitation letter was emailed to SEORL-CCC members with a single-use weblink explaining the nature of the survey and asking participants to answer according to their personal experiences. Willing respondents visited the electronic platform to take the survey. Questions were grouped by domains and displayed on three consecutive web pages. Participants were unable to proceed to the next domain if a question was left unanswered. Answers to the web-based survey were collected in the Cloud, downloaded, and evaluated by the principal researcher.

### Statistical analysis

Descriptive statistics were used to report results using SAS for Windows, version 9.4 (www.sas.com). Continuous variables were presented using mean, standard deviation (SD), median and interquartile range (IQR, first–third quartiles), while categorical variables were presented as counts and percentages. For some questions, more than one option was selected by the participants. In these cases, the percentage of each option was calculated. In addition, a subgroup analysis was carried out based on the existence or absence of a rhinology unit at the department where the ENT specialist works. Proportions between the two groups were compared using a Chi-squared test or Fisher's exact test (the latter when expected frequencies were lower than 20%) and a significance level of 5% (*p* < 0.05) was used. Data from participants that did not fully answer the survey were not included in the statistical analysis.

### Ethical considerations

The study was carried out according to the Declaration of Helsinki. Ethics committee approval was not required for the study. The survey was designed to avoid collecting personally identifiable information to maintain the anonymity of participants.

## Results

### Demographical data

A total of 180 ENT specialists responded to the survey; 127 participants (71%) from 15 autonomous regions in Spain answered all the survey questions and were included for statistical analysis (Online Resource Fig. 1). Fifty-three ENT specialists left the questionnaire unfinished, and these data were excluded. Participants had a mean ± SD age of 47.0 ± 11.4 years and were mainly employed at public hospitals (91%). Many of the surveyed ENT specialists had > 15 years of clinical experience (61%) and treated ≥ 15 patients with CRSwNP per month (71%). Table 1 shows the characteristics of the participating specialists.

**Table 1:** Demographic information of the ear, nose, and throat specialists

Number of participants	***N* = 127**
Mean ± SD age, years	47.0 ± 11.4
Healthcare working sector, *n* (%)	
Public sector	65 (51)
Private sector	11 (9)
Both	51 (40)
Working in an ENT department with a rhinology unit, *n* (%)	
Yes	85 (67)
No	42 (33)
Member of multidisciplinary asthma/united airway unit, *n* (%)	
Yes	62 (49)
No	65 (51)
Number of years of professional practice as an ENT specialist, *n* (%)	
< 5 years	23 (18)
5–15 years	27 (21)
> 15 years	77 (61)
Number of patients with CRSwNP treated per month, *n* (%)	
< 15 patients	37 (29)
15–30 patients	56 (44)
> 30 patients	34 (27)
Number of CRSwNP surgical interventions per month, *n* (%)	
None	11 (9)
< 5	62 (49)
≥ 5	54 (43)

*CRSwNP* chronic rhinosinusitis with nasal polyps, *ENT* ear, nose, and throat, *SD* standard deviation

### Assessment tools for evaluation of CRSwNP

**Fig. 1:**
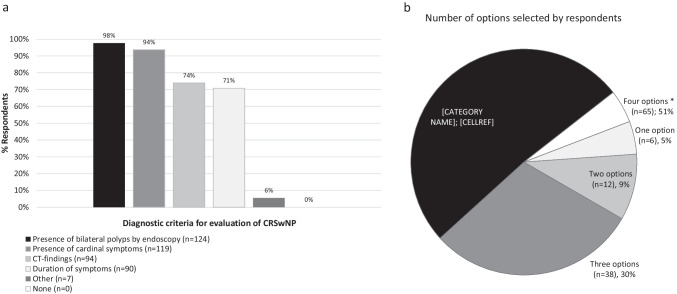
Diagnostic criteria used in routine clinical practice for CRSwNP evaluation. **a** Bars represent the proportion of ENT specialists that selected the answer (%). **b** Percentage of ENT specialists that selected one or more options simultaneously (%). *Five options, excluding the answer “none”. *CRSwNP* chronic rhinosinusitis with nasal polyps, *CT* computed tomography, *ENT* ear, nose, and throat

Ninety-eight percent of the surveyed ENT specialists indicated that bilateral polyps were diagnosed routinely using nasal endoscopy in patients with CRSwNP. Other commonly used methods of assessment were the presence of cardinal symptoms (94%), computed tomography (CT) (74%) and the duration of symptoms (71%). Only 30% of the ENT specialists used any combination of three of these criteria to confirm the diagnosis of CRSwNP, whereas 51% used four criteria (Fig. 1).

The preferred approach for assessing the severity of nasal obstruction, facial pain or olfactory impairment was to interview the patient about their symptoms (86%, 89% and 91% of respondents, respectively) and, to a lower extent, the use of visual analogue scale (VAS) scores (46%, 30% and 34%, respectively). Only a small proportion of ENT specialists (27%) indicated using smell tests, such as the University of Pennsylvania Smell Identification Test (UPSIT), Barcelona Smell Test (BAST)-24, Connecticut Chemosensory Clinical Research Center (CCCRC) or Sniffin' Sticks, to evaluate smell impairment. Severity of rhinorrhoea was evaluated by asking the patient about this symptom (88%) and by performing a nasal endoscopy evaluation (66%), whereas a VAS score evaluation for this symptom was only reported by 22% of ENT specialists. Most polled ENT specialists assessed QoL by asking the patient about the impact of the disease on their daily life (74%); however, less than half (45%) routinely used the 22-item sino-nasal outcomes test (SNOT-22). Endoscopic grading of nasal polyps was assessed by 97% of the ENT specialists surveyed, with the four-point Nasal Polyp Score (NPS) and the Lund-Kennedy score being the most common grading systems (60% and 32%, respectively) [Online Resource Figs. 2–7].

**Fig. 2:**
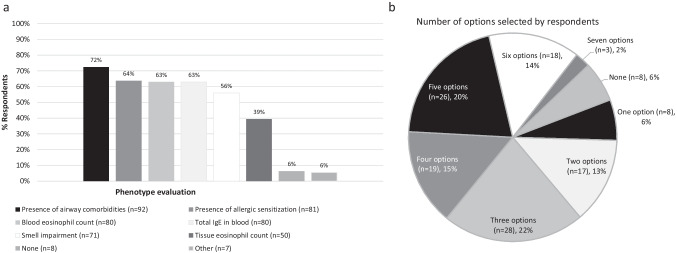
Characterization of inflammation in patients with CRSwNP. **a** Bars represent the proportion of ENT specialists that selected the answer (%). **b** Percentage of ENT specialists that selected one or more options simultaneously (%). *CRSwNP* chronic rhinosinusitis with nasal polyps, *ENT* ear, nose, and throat, *IgE* immunoglobulin E

Polled ENT specialists were presented with six items commonly used for evaluating the inflammatory profile of CRSwNP (Fig. 2). The most frequently selected item was evaluating airway comorbidities (72%), followed by allergic sensitization testing (64%) and evaluation of blood eosinophil levels (63%). A closer look at the answers indicated heterogeneous behavior in both the number of items selected and the combinations employed in daily practice (Fig. 2 and Online Resource Fig. 8). Almost half of the polled ENT specialists (43%) considered a blood eosinophil threshold of > 250 cel/μL as indicative of eosinophilic CRSwNP, whereas 25% would rather select a slightly higher threshold of > 300 cel/μL (Online Resource Fig. 9).

Most ENT specialists (76%) estimated that the fraction of patients with uncontrolled severe disease who visited their clinic was < 30%. Disease control was commonly assessed empirically based on the evaluation of patient-reported symptoms and disease evolution (59%) and/or by using the European Position Paper on Rhinosinusitis and Nasal Polyps 2020 (EPOS2020) control criteria (52%) (Online Resource Figs. 10–11).

### Medical and surgical management of CRSwNP

**Fig. 3:**
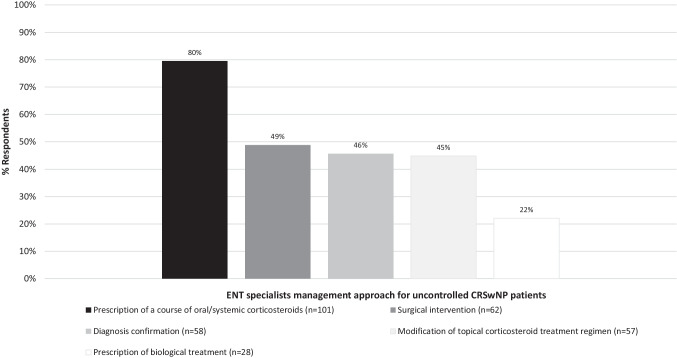
ENT specialist approach in patients with uncontrolled CRSwNP despite appropriate medical treatment. The bars represent the proportion of ENT specialists that selected the answer (%). *CRSwNP* chronic rhinosinusitis with nasal polyps, *ENT* ear, nose, and throat

In patients with uncontrolled CRSwNP, 80% of ENT specialists opted for prescribing a course of oral/systemic corticosteroids (Fig. 3 and Online resource Fig. 12). Two (54%) or three (38%) short courses of oral corticosteroids were prescribed per year before considering other management options (Online resource Fig. 13). Also, 49% of polled ENT specialists considered surgical intervention in patients who do not achieve disease control (Fig. 3). In this scenario, the most common approach for primary surgery was functional ESS (FESS) (60%) and extended ESS (EESS) involving frontal recess (38%) (Online resource Fig. 14).

**Fig. 4:**
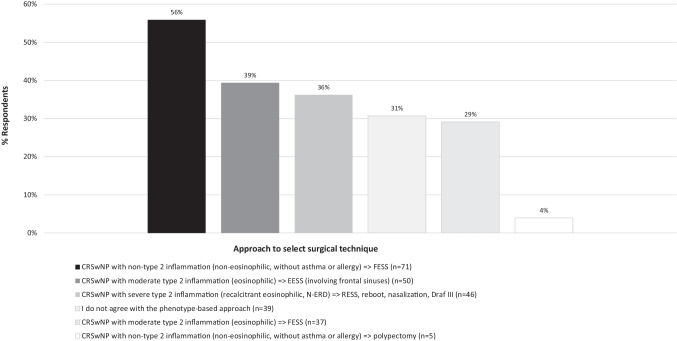
ENT specialist use of a phenotype-based approach to select a surgical technique. The bars represent the proportion of ENT specialists that selected the answer (%). *CRSwNP* chronic rhinosinusitis with nasal polyps, *ENT* ear, nose, and throat, *EESS* extended endoscopic sinus surgery, *FESS* functional endoscopic sinus surgery, *NSAID-ERD* non-steroidal anti-inflammatory drug-exacerbated respiratory disease, *RESS* radical endoscopic sinus surgery

Almost one-third of ENT specialists (31%) did not agree to follow a phenotype-guided surgical approach for CRSwNP. A large degree of heterogeneity was observed among the 68% ENT specialists who agreed with this approach, precluding the identification of underlying management trends (Fig. 4 and Online resource Fig. 15).

**Fig. 5:**
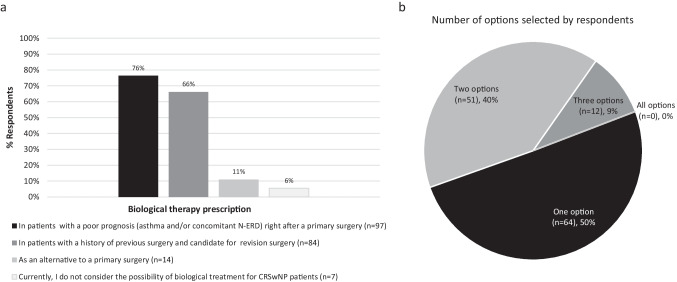
Scenario for initiation of biological treatment in patients with CRSwNP. **a** Bars represent the proportion of ENT specialists that selected the answer (%). **b** Percentage of ENT specialists that selected one or more options simultaneously (%). *CRSwNP* chronic rhinosinusitis with nasal polyps, *ENT* ear, nose, and throat, *NSAID-ERD* non-steroidal anti-inflammatory drug-exacerbated respiratory disease

Biological treatment was considered by 76% of ENT specialists after primary surgery in patients with comorbid asthma and/or non-steroidal anti-inflammatory drug-exacerbated respiratory disease [N-ERD]), as well as in patients with refractory disease who were already candidates for revision surgery (66%). Only 6% of ENT specialists did not consider biologics as a treatment option for patients with CRSwNP at the time of the survey (Fig. 5).

### Patient follow-up visits

For patients with severe CRSwNP, follow-up visits among most ENT specialists were scheduled every 3–4 months (73%) (Online resource Fig. 16). During these follow-up visits, 75% of ENT specialists proactively asked their patient if they had self-medicated with oral corticosteroids to manage CRSwNP since the last visit, while 89% interviewed patients about the intake of oral corticosteroids for managing other diseases since the last visit (Online resource Figs. 17–18). Almost all ENT specialists (98%) recommended daily intranasal corticosteroids and saline irrigations and scheduled an outpatient appointment with their patients after a CRSwNP surgical procedure (Online resource Fig. 19).

### Stratified analysis based on the availability of a rhinology unit within the ENT department

From the total sample of 127 ENT specialists who completed the survey, 85 (67%) worked at an ENT department with an available rhinology unit, whereas 42 (33%) worked in an ENT department that did not have a rhinology unit. A larger proportion of ENT specialists working at a rhinology unit reported the use of scoring systems to evaluate nasal obstruction severity (VAS: 53% vs 33%, *p = *0.0371), smell impairment (VAS: 41% vs 19%, *p = *0.0132, smell tests: 36% vs 7%, *p = *0.0004), the impact on QoL (SNOT-22: 56% vs 21%, *p = *0.0002), and polyp size (NPS: 66% vs 48%, *p = *0.04; Lund–Kennedy score: 42% vs 12%, *p = *0.0006). A larger proportion of ENT specialists at rhinology units reported using tissue eosinophilia as a criterion for evaluation of local eosinophilic inflammation (49% vs 19%, *p = *0.001). Similar results were observed when evaluating disease control using the EPOS2020 control tool (64% vs 29%, *p = *0.0002). Preference for different primary surgery approaches seemed to differ based on the availability of a rhinology unit, with FEES and EESS involving opening of the frontal recess selected by 48% and 49% versus 83% and 14% of ENT specialists with versus without an available rhinology unit, respectively (*p* < 0.0001). The use of biological therapies also seemed to be adopted more frequently among ENT specialists at rhinology units, with only 2% not currently considering the use of biological therapies versus 12% of ENT specialists without an available rhinology unit (*p = *0.039).

## Discussion

This online survey provides a key insight into the current perceptions and practices of ENT specialists managing patients with CRSwNP in Spain. Overall, the responses of the ENT specialists showed a good consistency with international guidelines, such as EPOS2020 [2], with more than 90% using cardinal symptom assessment or endoscopy as criteria for diagnosis. Symptom duration was used as a criterion by 71% of ENT specialists. While one might expect this number to be higher, the specific time restriction for the duration of symptoms (> 12 weeks) was not included in the questionnaire. It is possible that some ENT specialists did not select this item because they assumed the question to be vaguely phrased or that the symptom duration of > 12 weeks was implied. Nasal congestion, facial pain and smell impairment were mainly evaluated by interviewing the patient. Only 30–46% of the ENT specialists used VAS in routine clinical practice; rhinorrhoea was mainly evaluated by nasal endoscopy. International guidelines recommend using patient-reported outcomes (PROs), such as VAS, to quantify the severity of CRSwNP symptoms and facilitate their assessment over time. Rhinorrhoea can be simultaneously evaluated by ENT specialists when grading nasal polyps by endoscopy, a fact that might explain the preference for this technique over VAS. In fact, grading systems, such as the Lund-Kennedy score, used by almost one-third of polled ENT specialists, incorporates scoring systems for rhinorrhoea and edema.

QoL was routinely evaluated by patient interview (74%), and to a much lesser extent, by using SNOT-22 (45%). The importance of QoL assessment in the management of patients with CRSwNP has been demonstrated, and SNOT-22 has been recommended for this purpose. SNOT-22 is also useful for determining surgical indication and biological therapy in patients with poorly controlled disease. However, SNOT-22 is a lengthy test and is less likely to be implemented in resource-constrained environments, such as ENT departments without rhinology units. More time-saving options, like online tests, might help increase SNOT-22 use in clinical practice.

Most ENT specialists in this study (94%) have integrated the characterization of the inflammatory profile into their daily practice. This scenario is also reflected by the rapid uptake of the European Forum for Research and Education in Allergy and Airway Diseases (EUFOREA) and EPOS2020 blood eosinophil threshold recommendations for evaluation of eosinophilic inflammation, with more than two-thirds of polled ENT specialists (69%) selecting a cut-off of > 250/μL or > 300/μL. These results suggest that CRSwNP patient phenotyping may be routinely carried out, paving the way to identify patients who may benefit from biological treatments. In fact, the Spanish ENT specialists in this study currently regarded monoclonal antibody therapy as a potential treatment option in refractory CRSwNP after the failure of primary surgery. The adoption of these therapies prior to primary surgery is a topic of current debate, which might explain the low proportion of ENT specialists in this study who indicated they would consider it and instead opted for a surgical intervention. In this scenario, FESS and EESS with frontal recess opening were the most commonly chosen techniques. It is worth noting that none of the EMA approved biological therapies (dupilumab, omalizumab and mepolizumab) were reimbursed in Spain at the time of writing this manuscript. Therefore, ENT specialist answers about the timing and the patient profile candidate to biologic treatment were unlikely influenced by national reimbursing restrictions as they are yet to be defined.

A more diverse management trend was observed when using a phenotype-driven approach for CRSwNP surgery. The observed heterogeneity may be due to the fact that phenotype-driven CRSwNP surgery is a novel proposal yet to be widely endorsed and validated by the ENT community [14]. Current guidelines restrict the use of systemic corticosteroid to no more than two short courses per year in patients with severe uncontrolled disease. While the efficacy of this therapy is widely acknowledged, the detrimental side effects of systemic exposure to steroids in the short and long term are equally demonstrated in the literature [15]. Our results suggest that ENT specialists monitor to some extent the number of prescribed courses and keep track of patients' self-medication, along with the consumption of systemic steroids due to other inflammatory conditions such as asthma [16]. This latter behavior suggests a certain degree of multidisciplinary management with other specialties, such as pulmonologists and allergists.

ENT specialists working in a department with a rhinology unit were more likely to implement a systematic evaluation based on PROs, tests, and objective tools. There may be multiple, diverse, and intertwined explanations for these findings. For instance, large departments with rhinology units might have more available resources, including dedicated nursing staff. Also, having rhinology-specialized staff favors a more systematic unit organization, streamlining patient management and waiting lists. In some cases, recurrent specialized CRSwNP outpatient consultations are scheduled, where patients with severe disease undergo a full evaluation in a single day. We acknowledge that any rationale offered for this observed trend should be evaluated cautiously, and further validation with a larger sample size is required.

At the time of writing this manuscript, a survey describing trends in managing patients with CRSwNP by the Young Otolaryngologists of International Federation of Oto-rhino-laryngological Societies (YO-IFOS) group had previously been published [9]. Both YO-IFOS and the current survey displayed a similar proportion of specialists evaluating airway comorbidities (72% vs 72%, respectively), allergic sensitization (66% vs 64%), blood eosinophil counts (70% vs 63%), tissue eosinophil counts (41% vs 40%) or SNOT-22 use (57% vs 45%). On the other hand, differences in patient management can also be observed between the current survey versus the YO-IFOS survey, such as the elapsed time between follow-up visits (3–4 months vs 6 months) or the use of score systems to grade the size of nasal polyps (97% vs 60%). However, it is unclear whether the results can be compared between surveys, particularly considering factors such as the demographic and geographical disparity between both surveyed samples, differences in the phrasing of survey questions, differences in question interpretation by the polled specialists, or differences in region-dependent availability of certain medications. Trends in the evaluation of symptom severity between both samples could not be carried out as this aspect was not explicitly surveyed by the YO-IFOS group.

Some limitations in the present study are noteworthy when interpreting the results. Our work was limited by its sample size and asymmetric representation of different Spanish regions, restricting our ability to map and compare specific regional behaviors. Additionally, ENT specialist participation in this survey was purely voluntary, and data from specialists who started the survey but did not finish it were discarded. There are over 2000 ENT specialists in Spain, but those interested in CRSwNP disease are a fraction of this number, so non-response bias was unavoidable. The observed response rate and the number of participating ENT specialists was similar to previous surveys carried out by our society and others [18, 19]. We considered the “Rhinology Unit” to be a unit with adequate infrastructure and staff to perform the assigned functions. Since we did not provide this detailed definition to survey participants, interpretation bias may have affected the stratified analysis.

In contrast, one of the strengths of this study is that it is the largest survey undertaken among ENT specialists in Spain on current practices and perceptions of CRSwNP patient care, providing critical insights into the current strengths and areas of optimization. From our point of view, several areas of intervention can be extracted from the results. First, ENT specialist knowledge of the latest evidence and current CRSwNP guidelines seems essential for gradually incorporating evidence-based recommendations into real-life clinical practice and optimizing patient care [17]. This approach suggests a subsequent need for enhancing the awareness of guideline-recommended clinical management among the ENT specialist community in Spain and implementing disease-specific scientific forum discussions to debate the latest published evidence in the pathology of CRSwNP. In this regard, we find of paramount importance to create specific initiatives for promoting the use of PROs and objective clinical evaluation tools to assess disease control and severity. Increasing the use of such tools among ENT specialists would likely result in a more precise management of patients with CRSwNP.

## Conclusion

This survey revealed that Spanish ENT specialists showed a trend toward incorporating new concepts and management proposals based on patient phenotyping, characterization of inflammation and the use of biological therapies. Patient reported outcomes and objective clinical evaluation tools, such as symptom VAS, SNOT-22 or smell tests, showed a poor uptake in routine clinical practise. Specific training programmes could improve awareness and adherence to the guidelines by ENT specialists, further optimizing the management of patients with CRSwNP.

### Supplementary Information

Below is the link to the electronic supplementary material.

Supplementary file1 (DOCX 870 KB)

## Data Availability

Anonymised data are available from the authors upon request.
