# Supplementary material for: Management of patients with chronic rhinosinusitis with nasal polyps in Spain: learnings from a nationwide survey of otorhinolaryngologists
Source: Eur Arch Otorhinolaryngol. 2023 Sep 1;281(1):227–35. doi: 10.1007/s00405-023-08185-5 (PMC10764407; doi:10.1007/s00405-023-08185-5)
Supplement: Supplementary file 1 — Supplementary file1 (DOCX 870 KB) [file 405_2023_8185_MOESM1_ESM.docx]

# Online resource figures

## Online resource Fig. 1


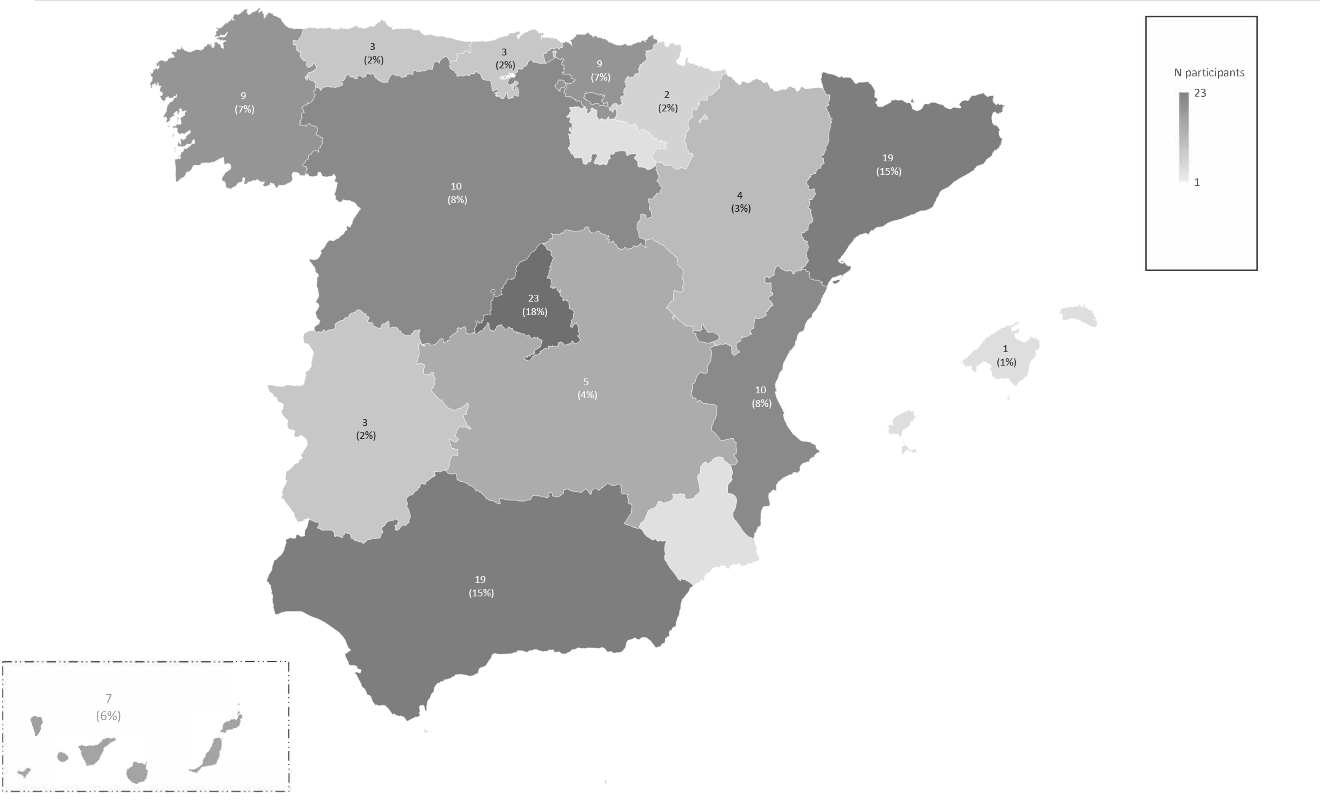


Geographical distribution of ENT specialists surveyed. The map represents the number (%) of ENT specialists surveyed for each autonomous Spanish region. The gradient of colour is proportional to the frequency of observations: the darker the colour, the higher the frequency. *ENT* ear, nose, and throat

## Online resource Fig. 2


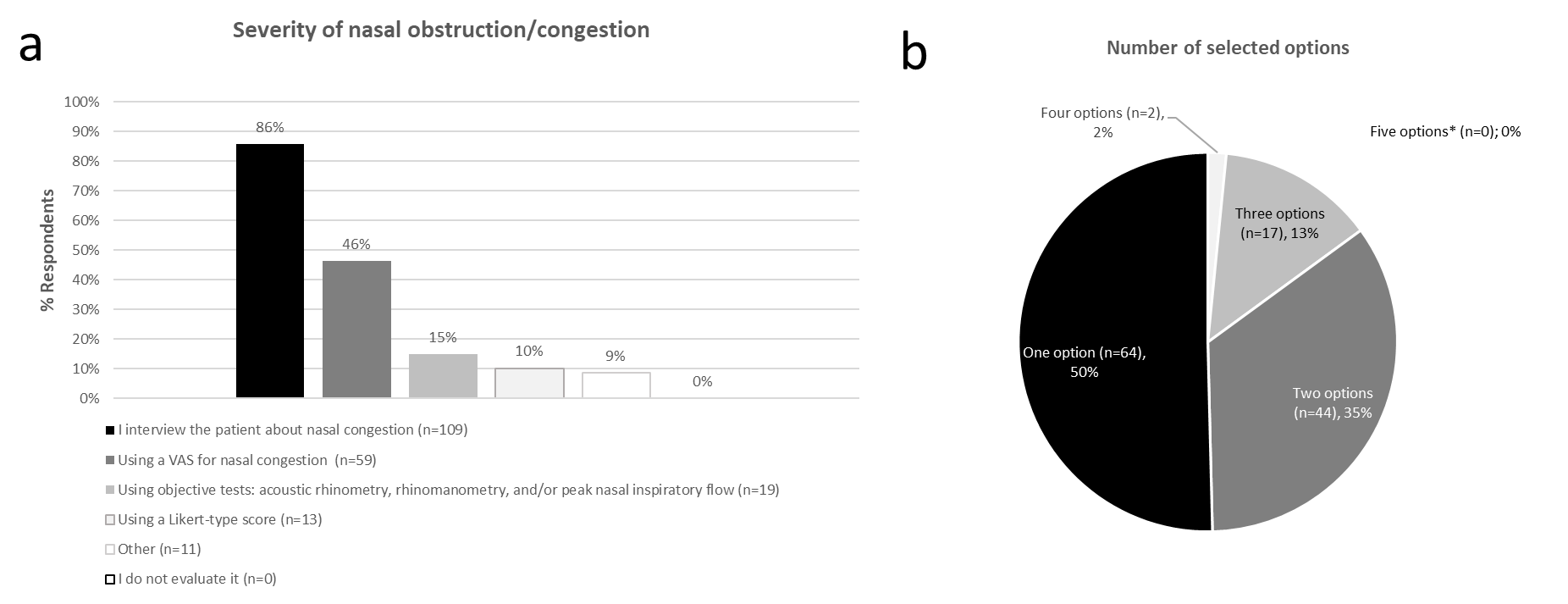


Evaluation of severity of nasal obstruction/congestion in routine clinical practice. **(a)** Bars represent the proportion of ENT specialists that selected the answer (%). **(b)** Percentage of ENT specialists that selected one or more options simultaneously (%) *Five options excluding the answer “I do not evaluate it”. *ENT* ear, nose, and throat, *VAS* visual analogue scale

## Online resource Fig. 3


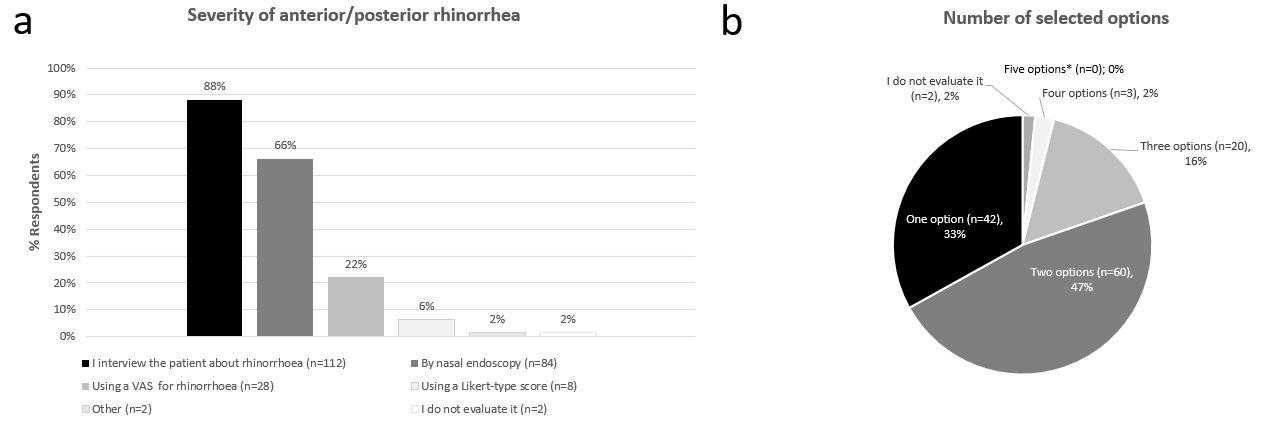


Evaluation of severity of anterior/posterior rhinorrhoea in routine clinical practice. **(a)** Bars represent the proportion of ENT specialists that selected the answer (%). **(b)** Percentage of ENT specialists that selected one or more options simultaneously (%) *Five options excluding the answer “I do not evaluate it”. *ENT* ear, nose, and throat, *VAS* visual analogue scale

## Online resource Fig. 4


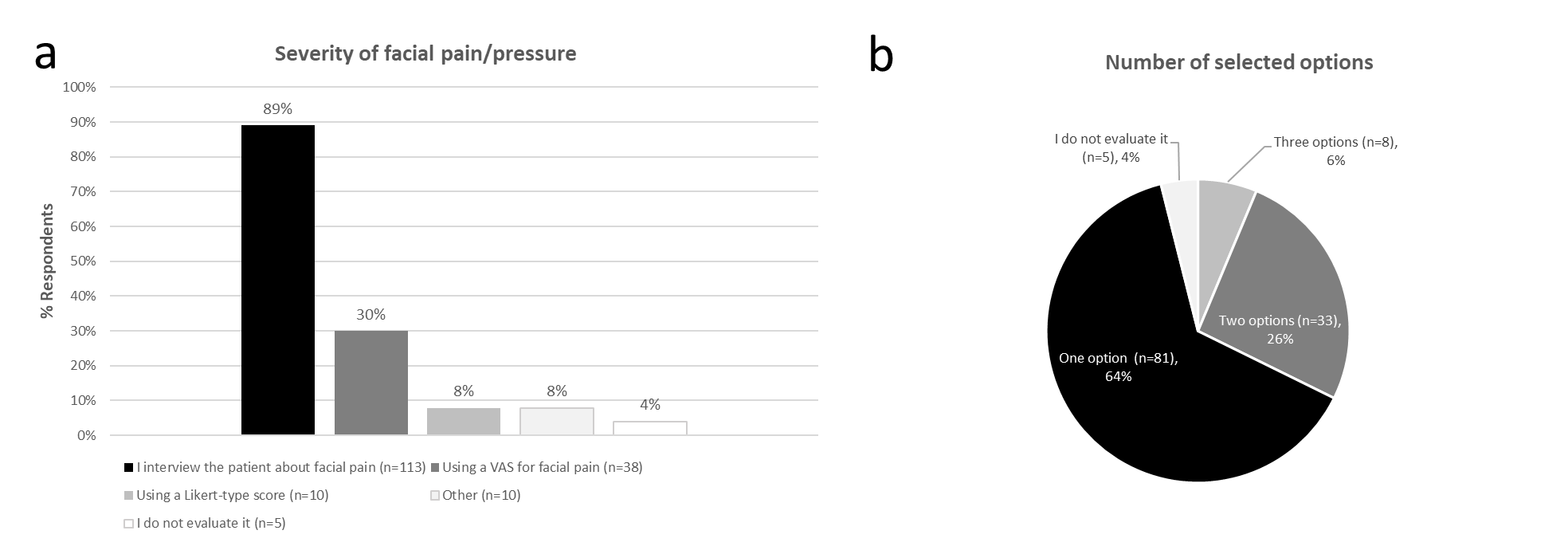


Evaluation of severity of facial pain or pressure in routine clinical practice. **(a)** Bars represent the proportion of ENT specialists that selected the answer (%). **(b)** Percentage of ENT specialists that selected one or more options simultaneously (%) *Four options excluding the answer “I do not evaluate it”. *ENT* ear, nose, and throat, *VAS* visual analogue scale

## Online resource Fig. 5


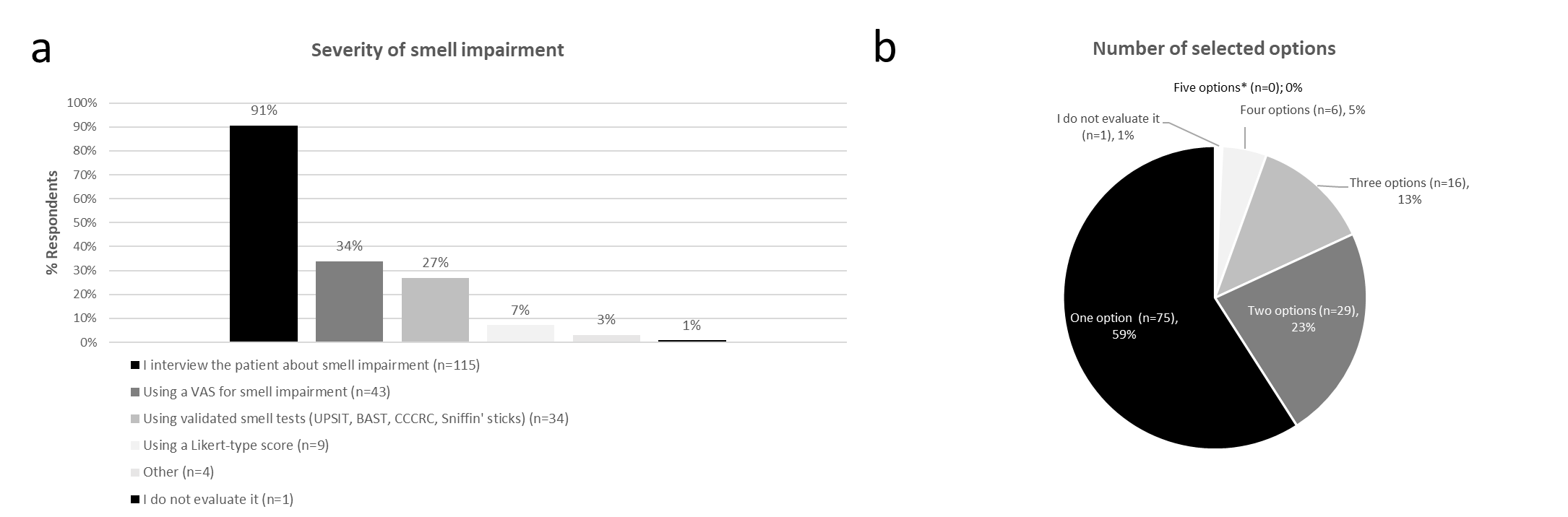


Evaluation of severity of smell impairment in routine clinical practice. **(a)** Bars represent the proportion of ENT specialists that selected the answer (%). **(b)** Percentage of ENT specialists that selected one or more options simultaneously (%) * Five options excluding the answer “I do not evaluate it”. *BAST* Barcelona Smell Test, *CCCRC* Connecticut Chemosensory Clinical Research Center, *ENT* ear, nose, and throat, *UPSIT* University of Pennsylvania Smell Identification Test, *VAS* visual analogue scale

## Online resource Fig. 6


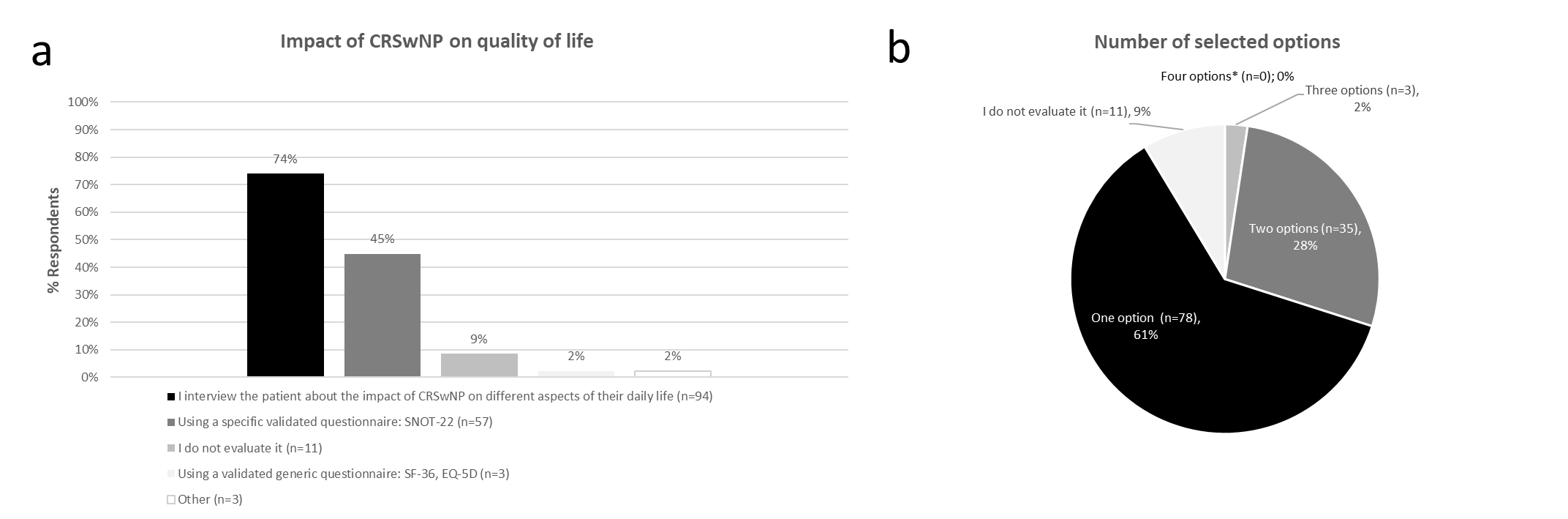


Evaluation of impact of CRSwNP on quality of life in routine clinical practice. **(a)** Bars represent the proportion of ENT specialists that selected the answer (%). **(b)** Percentage of ENT specialists that selected one or more options simultaneously (%). *Four options excluding the answer “I do not evaluate it”. *CRSwNP* chronic rhinosinusitis with nasal polyps, *EQ-5D* Euro-QoL-5, *SNOT-22* 22-item Sino-Nasal Outcomes Test, *SF-36* 36-Item Short Form Health Survey

## Online resource Fig. 7


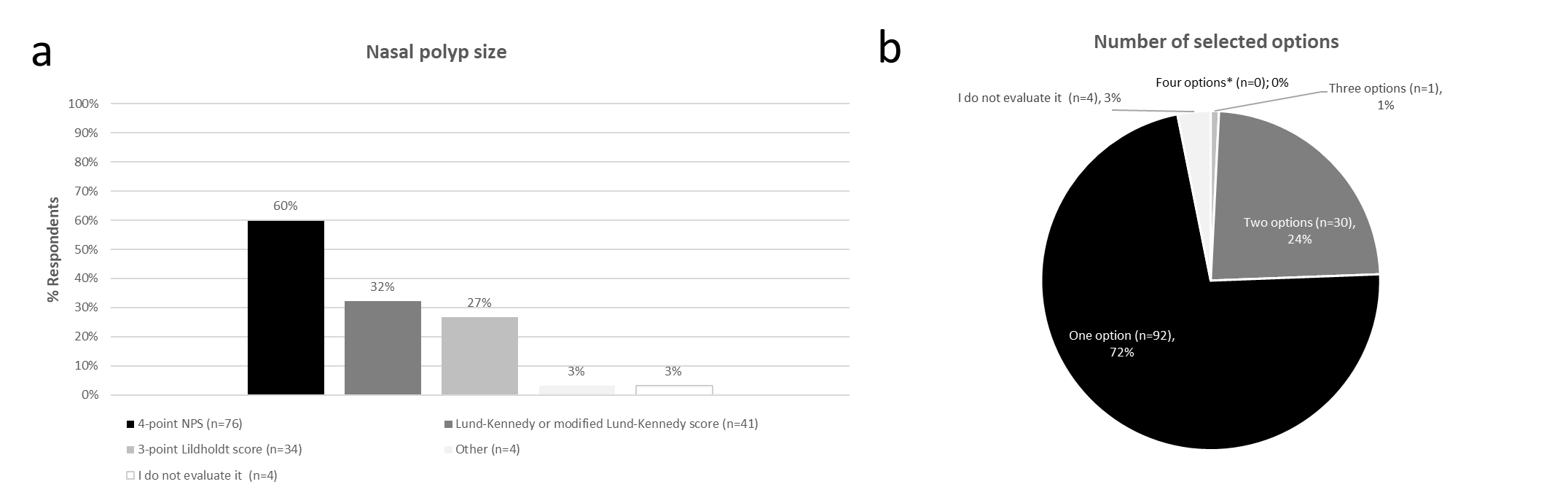


Evaluation of size of nasal polyps in patients with CRSwNP in routine clinical practice. **(a)** Bars represent the proportion of ENT specialists that selected the answer (%). **(b)** Percentage of ENT specialists that selected one or more options simultaneously (%). *Four options excluding the answer “I do not evaluate it”. *CRSwNP* chronic rhinosinusitis with nasal polyps, *ENT* ear, nose and throat, *NPS* nasal polyp score

## Online resource Fig. 8


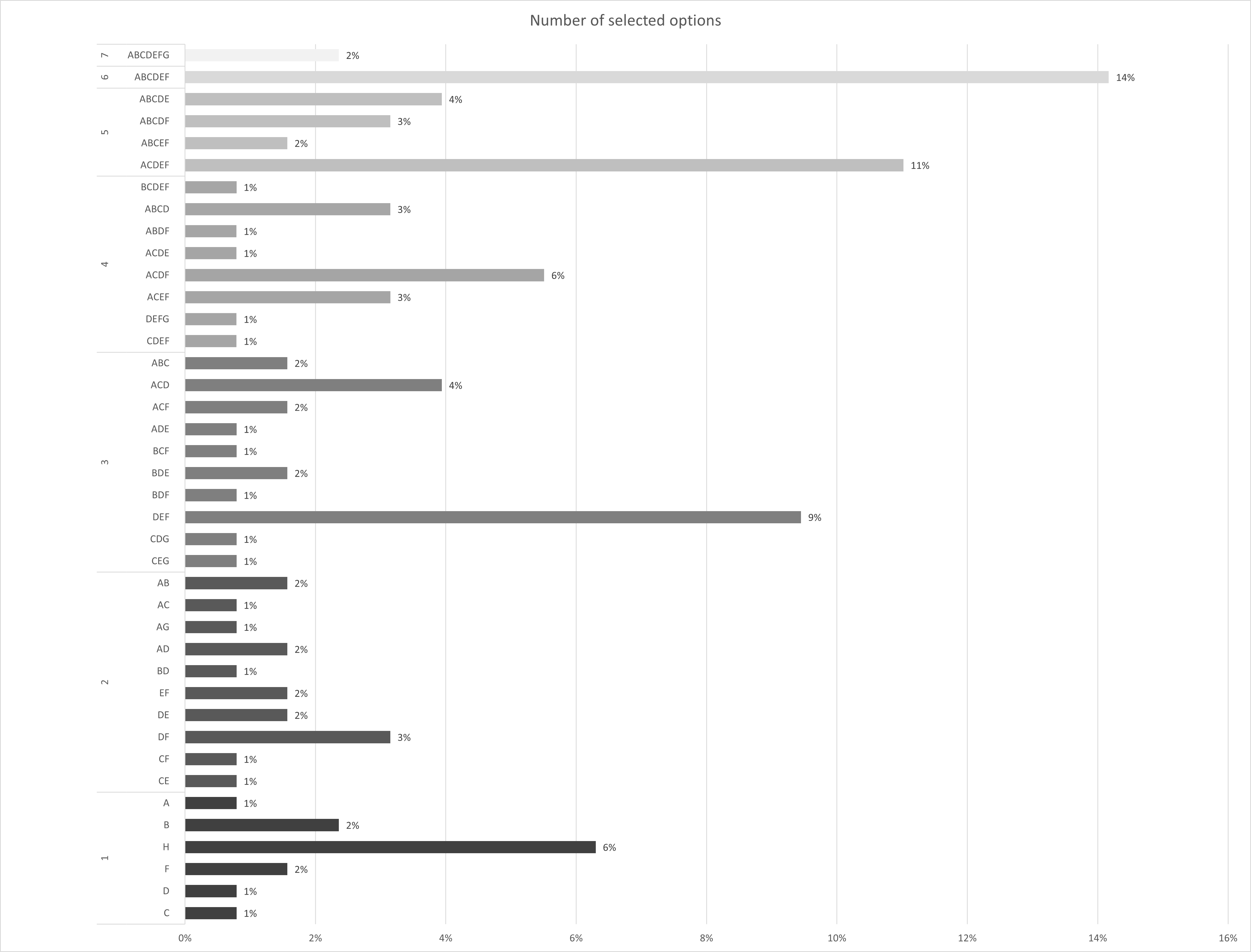


Tests used to define the inflammation profile in patients with CRSwNP. Bars represent the proportion of ENT specialists that selected the answer (%). A: Blood eosinophil count; B: Tissue eosinophil count; C: total IgE in blood; D: presence of airway comorbidities; E: smell impairment; F: Presence of allergic sensitization; G: Other; H: None. *CRSwNP* chronic rhinosinusitis with nasal polyps, *ENT* ear, nose and throat, *IgE* immunoglobulin E

## Online resource Fig. 9


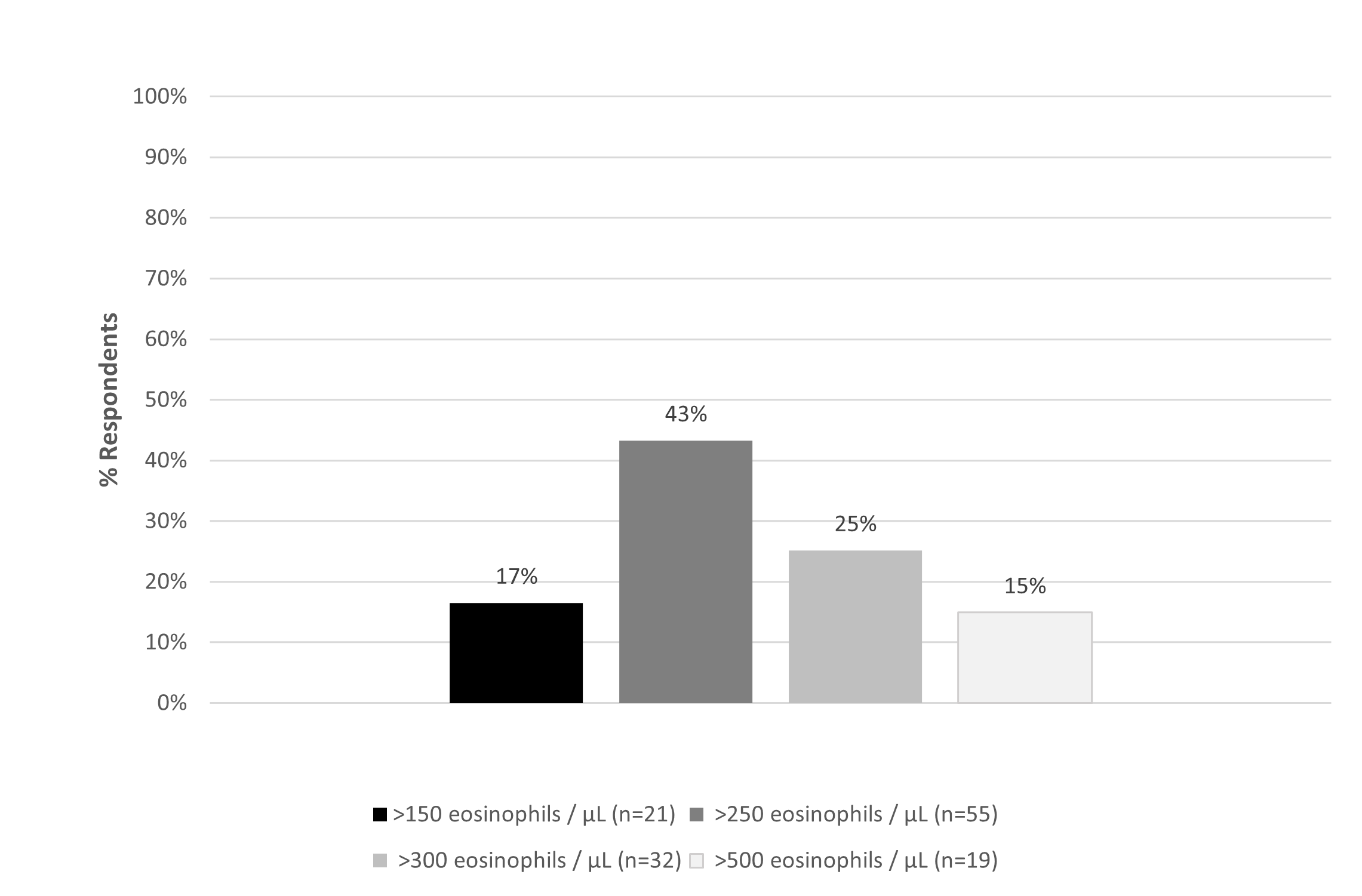


Blood eosinophil count threshold to consider a diagnosis of eosinophilic CRSwNP. Bars represent the proportion of ENT specialists that selected the answer (%)

## Online resource Fig. 10


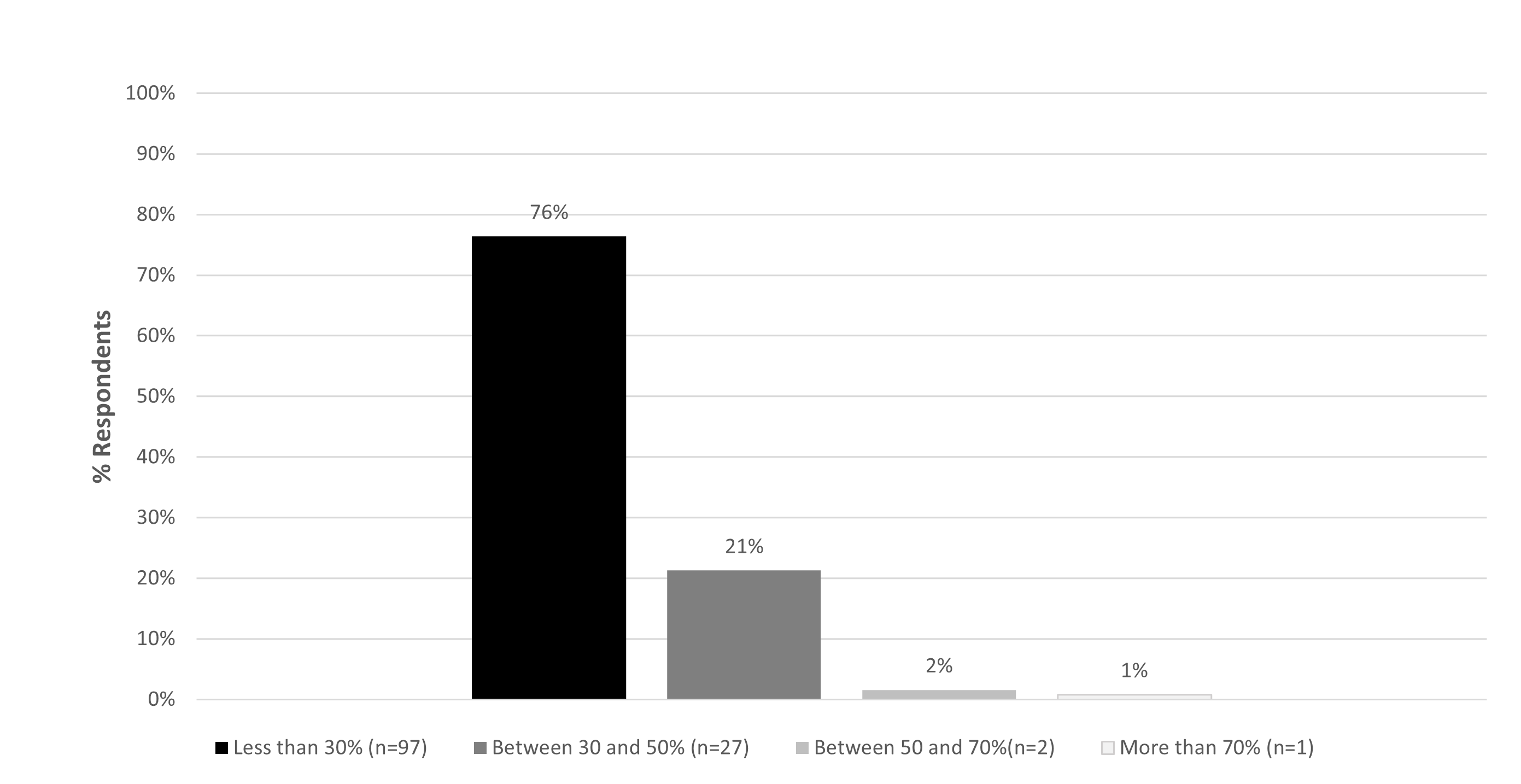


Proportion of patients with CRSwNP classified as “severe uncontrolled” in routine clinical practice. Bars represent the proportion of ENT specialists that selected the answer (%). *CRSwNP* chronic rhinosinusitis with nasal polyps, *ENT* ear, nose and throat

**Online resource Fig. 11**
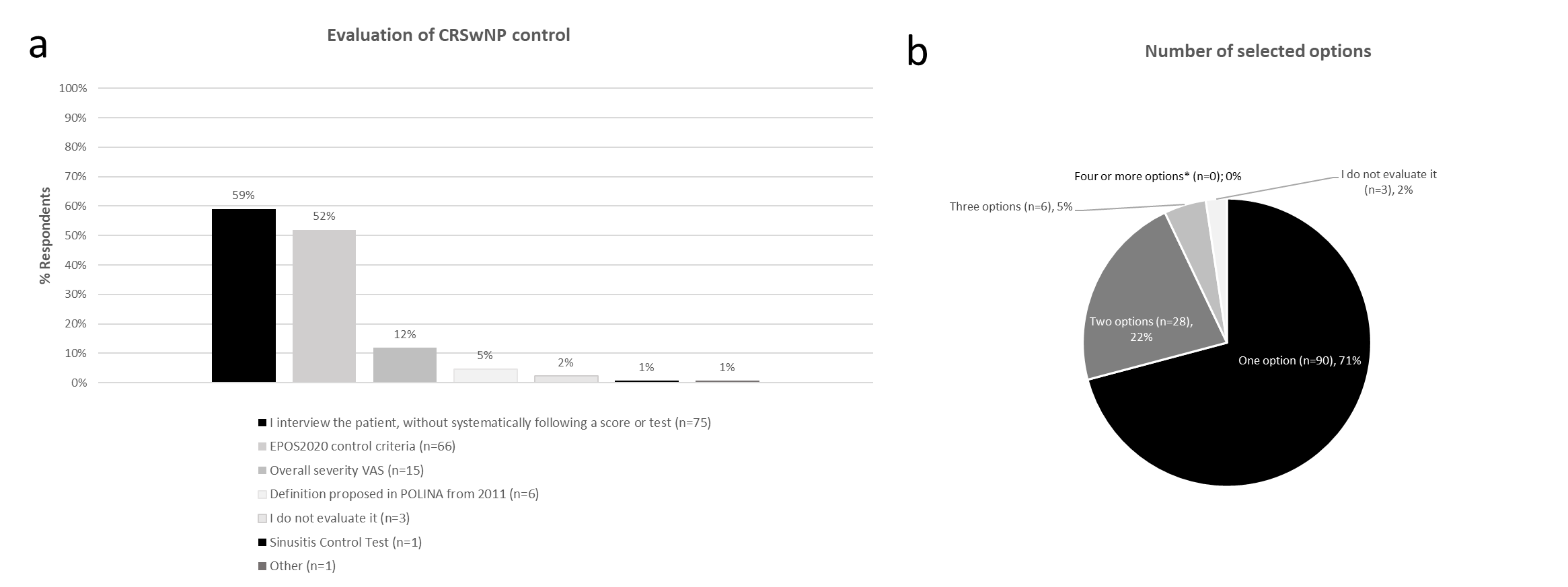


Evaluation of CRSwNP control in routine clinical practice. **(a)** Bars represent the proportion of ENT specialists that selected the answer (%). **(b)** Percentage of ENT specialists that selected one or more options simultaneously (%). *Four or more options excluding the answer “I do not evaluate it”. *CRSwNP* chronic rhinosinusitis with nasal polyps, *ENT* ear, nose and throat, *EPOS2020* European Position Paper on Rhinosinusitis and Nasal Polyps 2020, *POLINA* Spanish Guide on Nasal Polyposis, *VAS* visual analogue scale

## Online resource Fig. 12


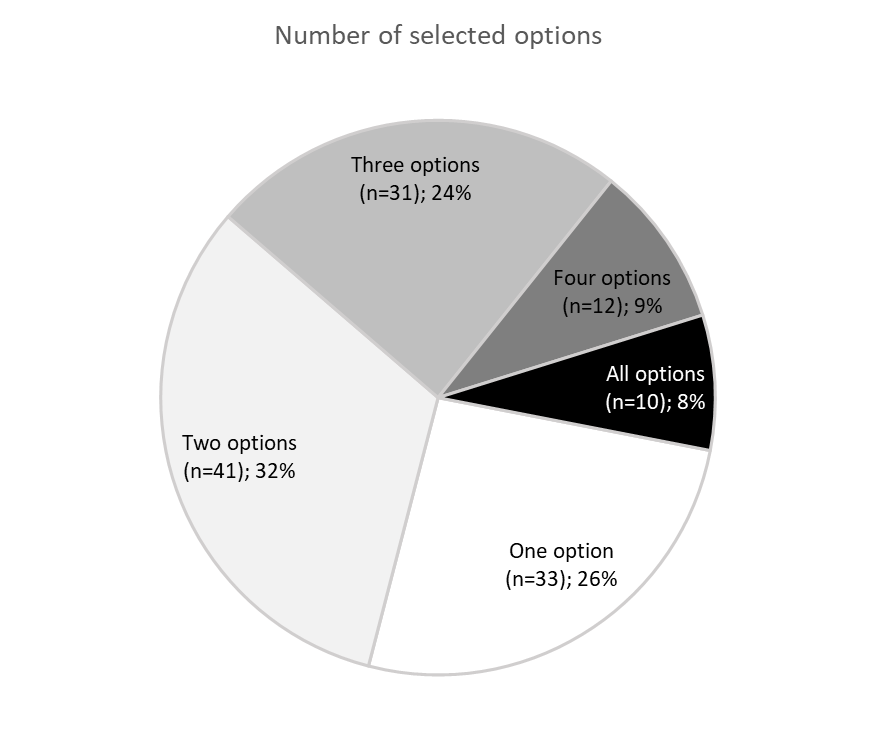


Approach of ENT specialists when a patient with CRSwNP shows lack of disease control despite appropriated medical treatment. The pie chart shows the percentage of ENT specialists that selected one or more options simultaneously (%) among the following: a course of oral/systemic corticosteroids; surgical approach; review and diagnosis confirmation; modification of topical corticosteroid treatment regimen; or treatment with a biologic agent. *CRSwNP* chronic rhinosinusitis with nasal polyps, *ENT* ear, nose and throat

## Online resource Fig. 13

**
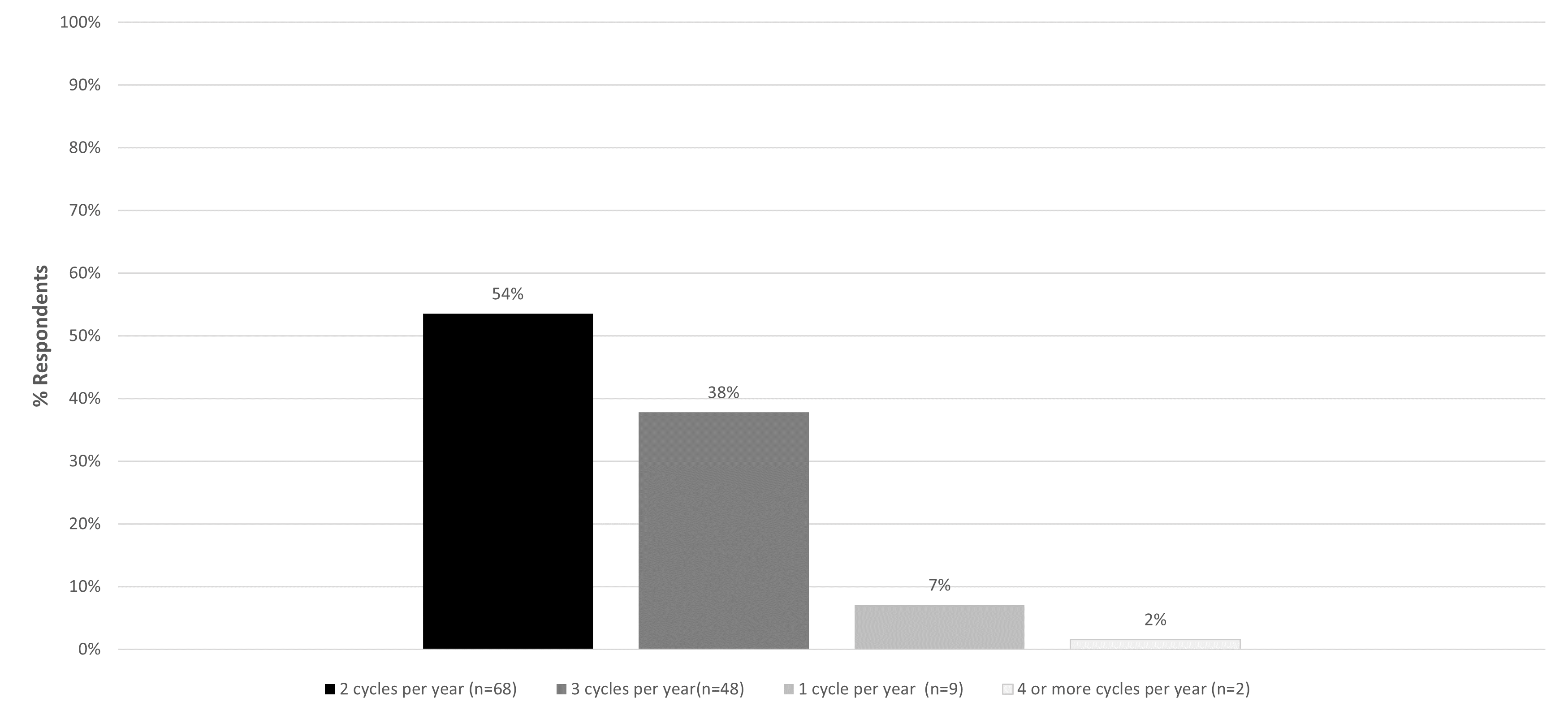
**

Maximum annual courses of oral corticosteroids prescribed to a patient with CRSwNP before considering other options (e.g. surgery or prescription of a biological treatment) . Bars represent the proportion of ENT specialists that selected the answer (%). *CRSwNP* chronic rhinosinusitis with nasal polyps, *ENT* ear, nose and throat

## Online resource Fig. 14


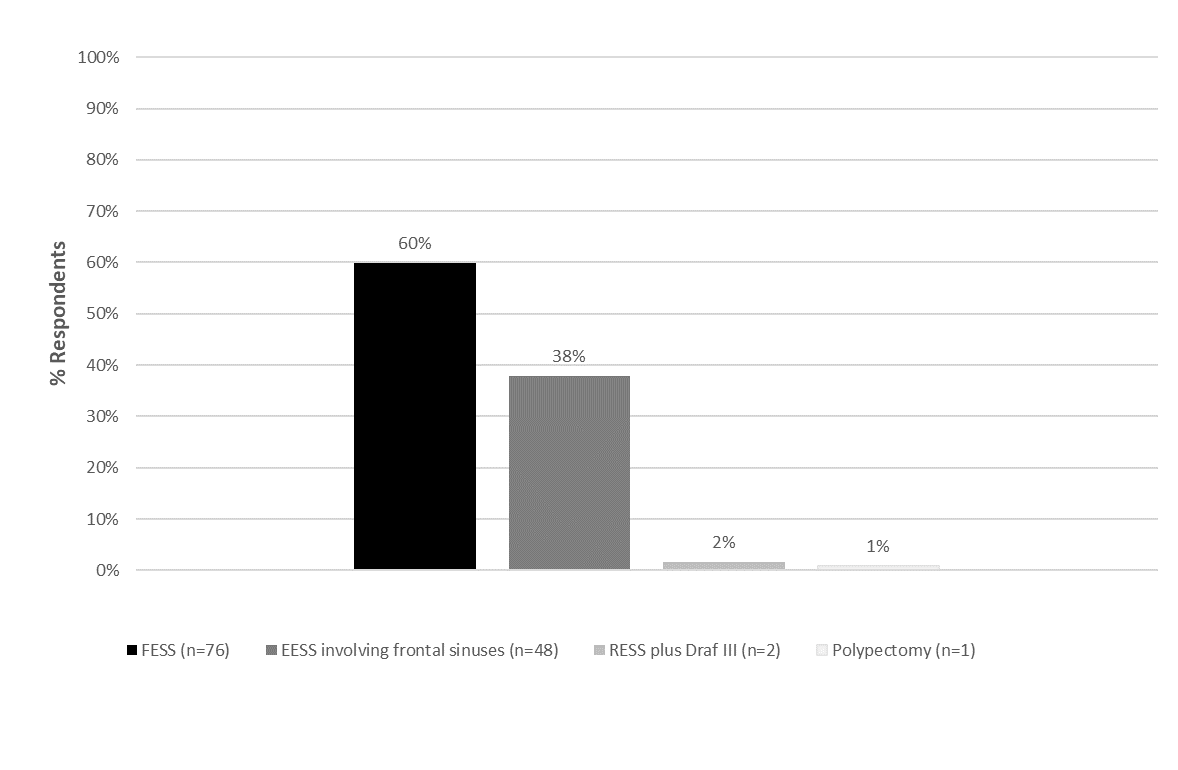


Type of primary surgery for CRSwNP most frequently performed in routine clinical practice. Bars represent the proportion of ENT specialists that selected the answer (%). *CRSwNP* chronic rhinosinusitis with nasal polyps, *ENT* ear, nose and throat, *EESS* extended endoscopic sinus surgery, *FESS* Functional endoscopic sinus surgery, *RESS* radical endoscopic sinus surgery

## Online resource Fig. 15


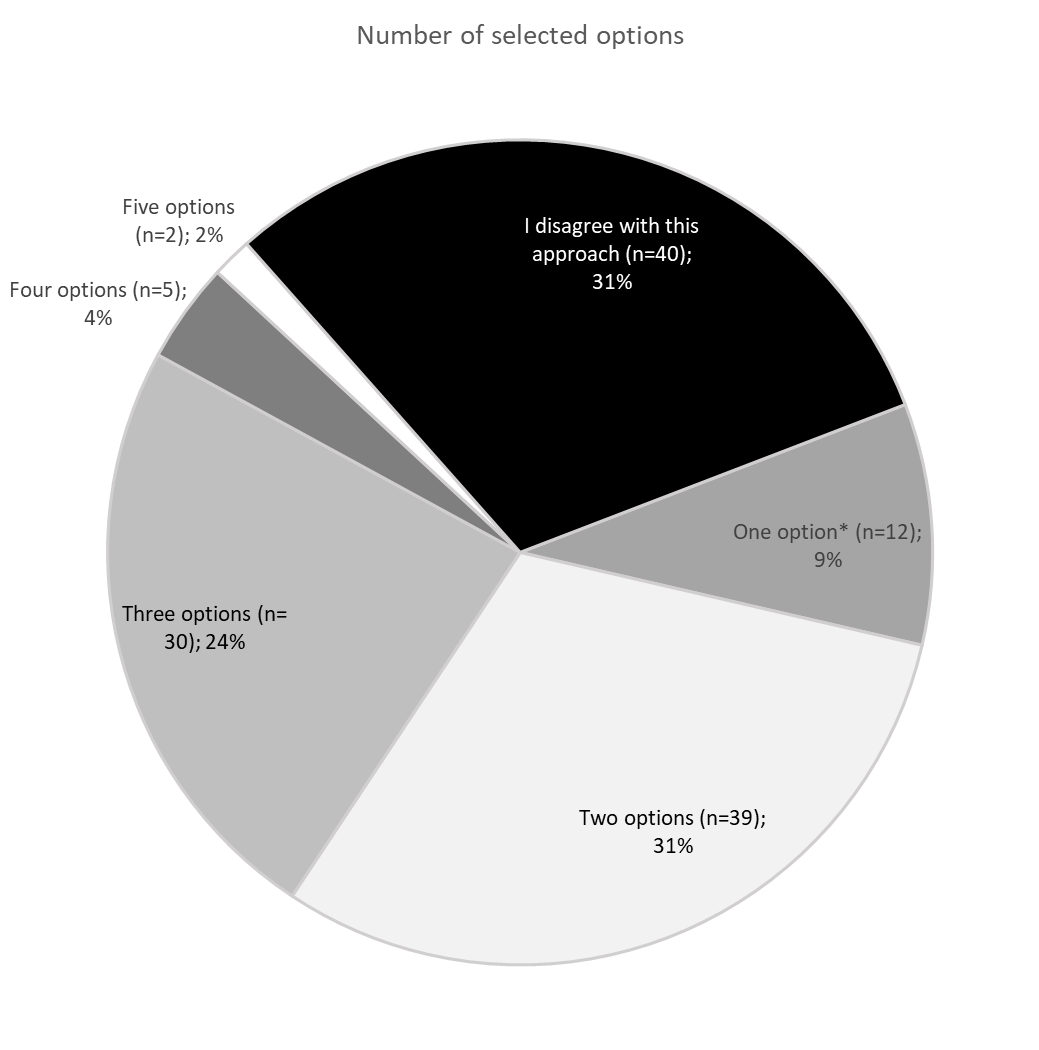


Use of a phenotype-based approach by ENT specialists to select a surgical technique. Percentage of the number of options chosen by the respondents (%). *; among the following: CRSwNP with non-type 2 => Functional endoscopic sinus surgery; CRSwNP non-type 2 => Polypectomy; CRSwNP with moderate type 2 inﬂammation=> Functional endoscopic sinus surgery; CRSwNP with moderate type 2 inﬂammation => Extended endoscopic sinus surgery involving frontal recess dissection; CRSwNP with severe type 2 inﬂammation => radical endoscopic sinus surgery, reboot technique, nasalization, DRAF III; External/open surgery; I disagree with this approach. * One option except "I disagree with this approach". CRSwNP* chronic rhinosinusitis with nasal polyps, *ENT* ear, nose and throat, *EESS* extended endoscopic sinus surgery, *FESS* functional endoscopic sinus surgery, *RESS* radical endoscopic sinus surgery

## Online resource Fig. 16


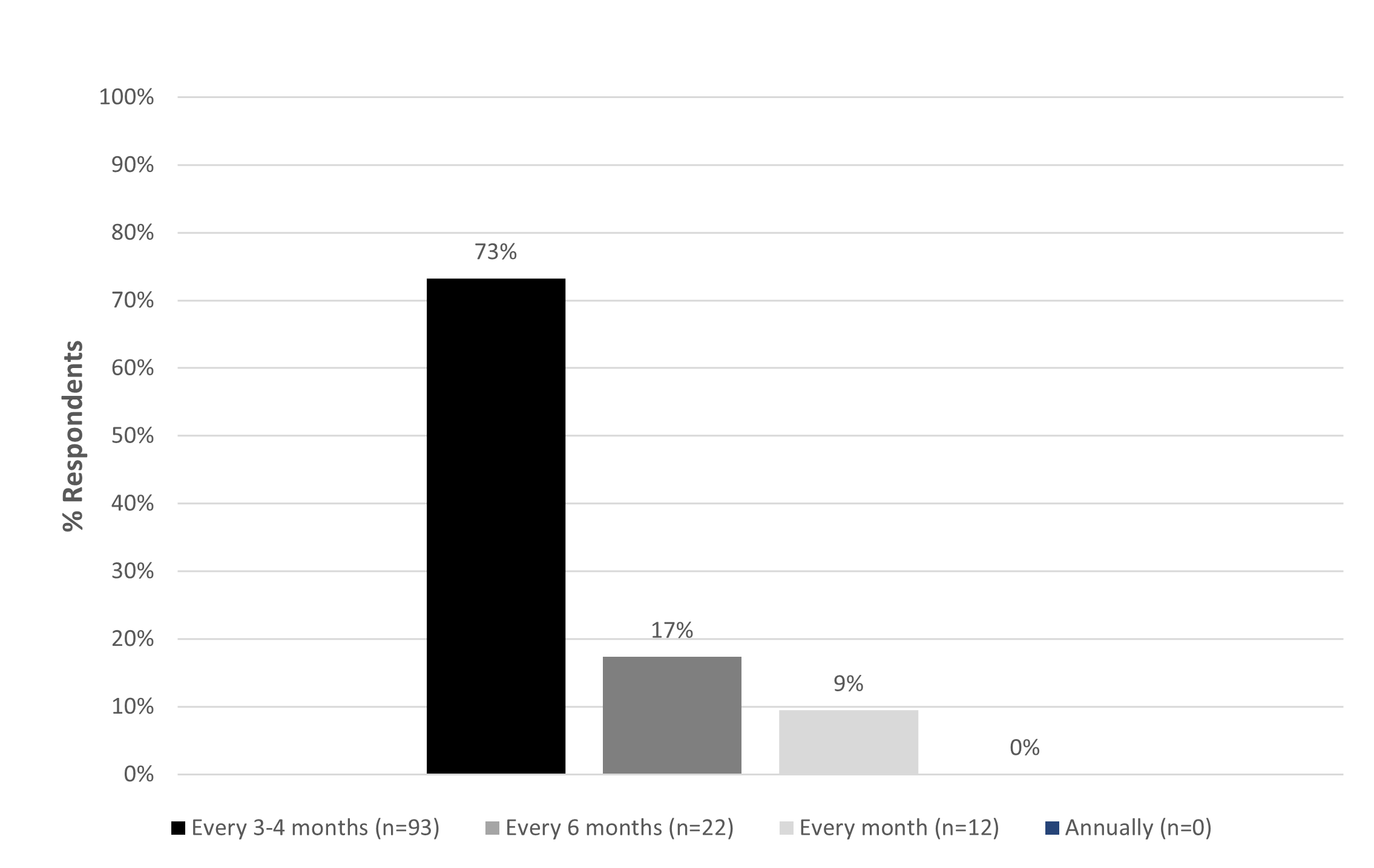


Frequency of follow-up visits in patients with severe CRSwNP. Bars represent the proportion of ENT specialists that selected the answer (%). *CRSwNP* chronic rhinosinusitis with nasal polyps, *ENT* ear, nose and throat

## Online resource Fig. 17


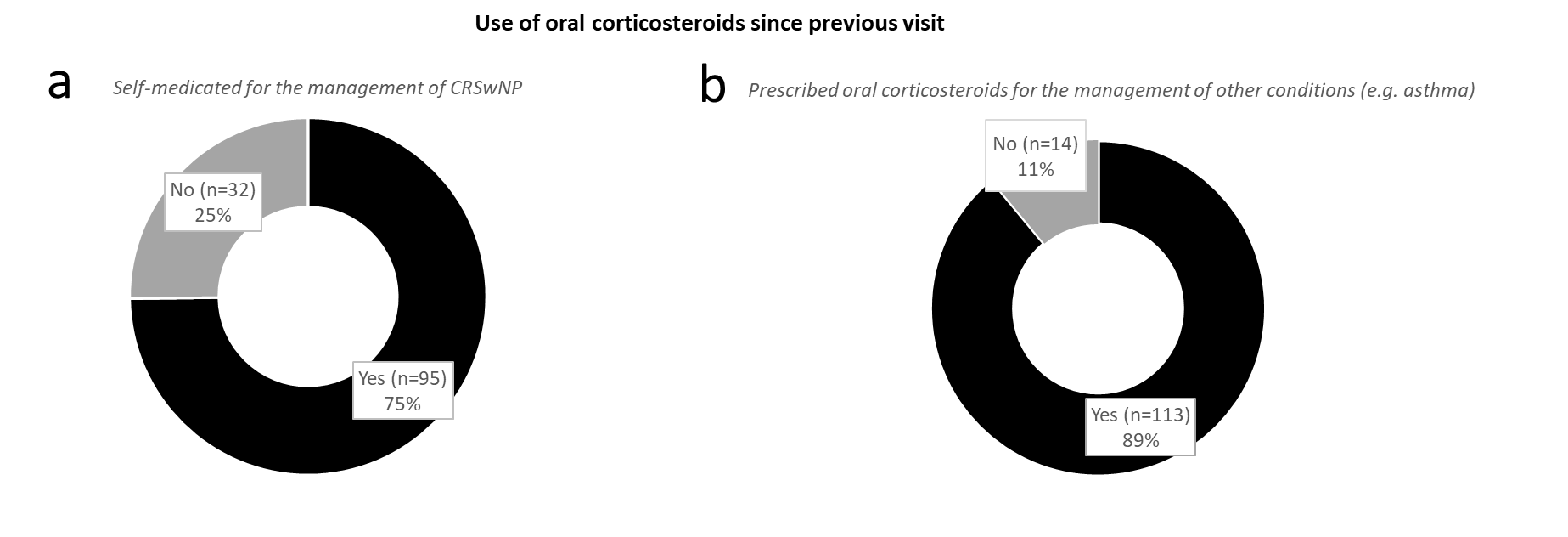


Questionnaire survey report on use of oral corticosteroids for the management of CRSwNP and/or other conditions. Question: During a follow-up visit, do you usually ask the patient…? **(a)** …If he/she has self-medicated with oral corticosteroids for the management of CRSwNP since the last visit. **(b)** …If he/she has been prescribed oral corticosteroids for the management of other conditions (e.g. asthma) since the last visit (%). *CRSwNP* chronic rhinosinusitis with nasal polyps, *ENT* ear, nose and throat

## Online resource Fig. 18


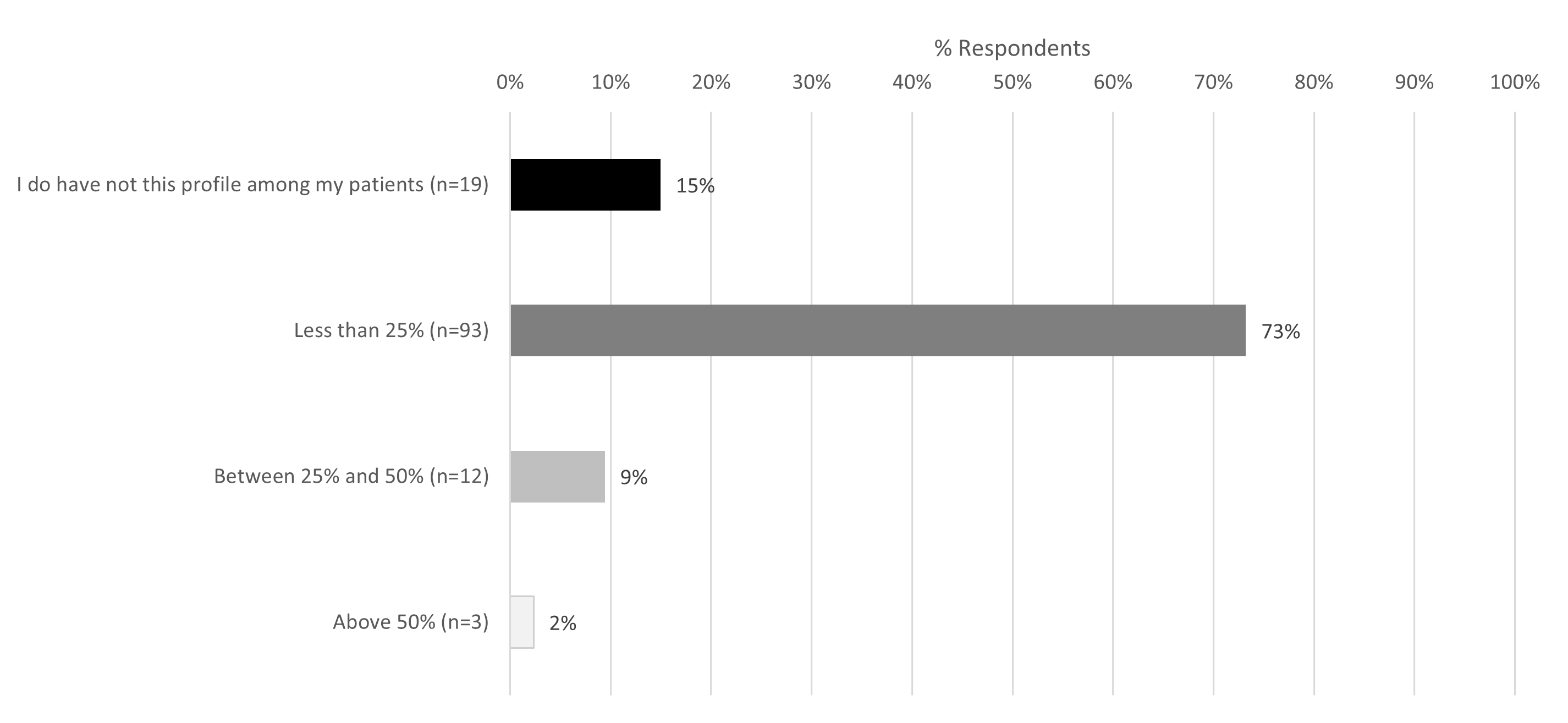


Proportion of patients with CRSwNP receiving maintenance treatment with oral corticosteroids for any condition. Bars represent the proportion of ENT specialists that selected the answer (%). *CRSwNP* chronic rhinosinusitis with nasal polyps, *ENT* ear, nose and throat

## Online resource Fig. 19


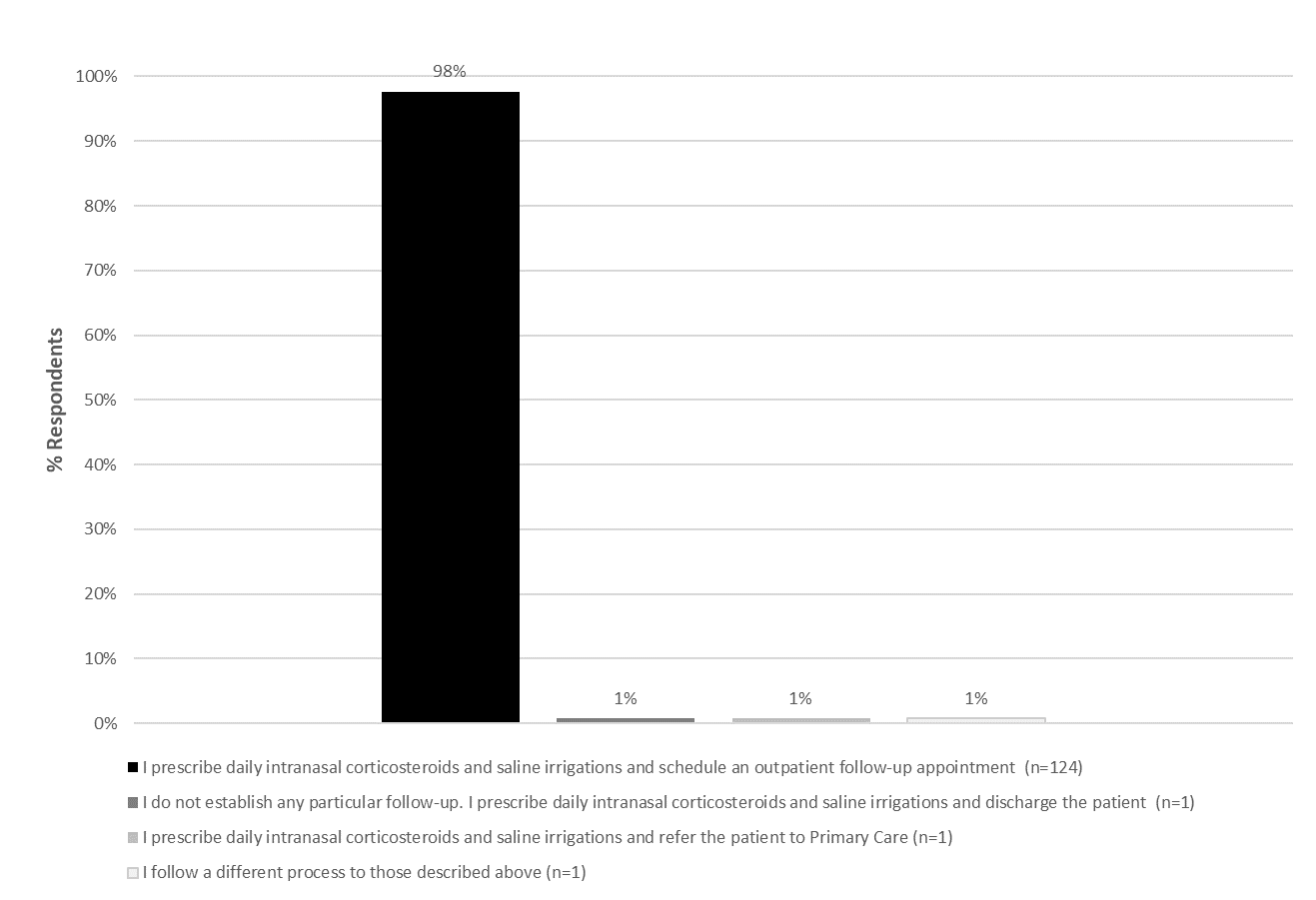


Patient follow-up after CRSwNP surgery. Bars represent the proportion of ENT specialists that selected the answer (%). *CRSwNP* chronic rhinosinusitis with nasal polyps, *ENT* ear, nose and throat.

## Appendix 1- Survey

Surveyed ENT demographic characteristics

- Age
- Autonomous Community of employment
- Public/private practice
- Do you work in a service that has a Rhinology unit?

Yes No

- Are you or any HCP of your service member of a multidisciplinary asthma/united airway unit in your hospital?

Yes No

- How long have you been practicing as an ENT specialist?

Less than 5 years. 5–15 years. More than 15 years.

- How many CRSwNP patients per month do you visit?

Less than 15 patients. 15–30 patients. More than 30 patients.

- How many CRSwNP surgical interventions do you perform per month?

None. Less than 5 per month. At least 5 per month.

Assessment tools for CRSwNP

1. Which of the following diagnostic criteria do you use in routine clinical practice to confirm a diagnosis of CRSwNP? [select all that apply]

- Guiding symptoms: nasal obstruction, anterior or posterior rhinorrhoea, smell impairment, facial pain.
- Duration of symptoms.
- Presence of bilateral polyps detected by nasal endoscopy.
- Computed tomography (CT) findings compatible with a diagnosis of CRSwNP.
- Other.
- None.

1. How do you evaluate the following endpoints in patients with CRSwNP? [select all that apply]

2.1. Nasal obstruction/congestion severity.

- I interview the patient about nasal congestion.
- Using a visual analogue scale (VAS) for nasal congestion.
- Using a Likert-type score.
- Using objective tests: acoustic rhinometry, rhinomanometry, and/or peak nasal inspiratory flow .
- Other.
- I do not evaluate it.

2.2. Anterior/posterior rhinorrhoea severity.

- I interview the patient about rhinorrhoea.
- Using a visual analogue scale (VAS) for rhinorrhoea.
- Using a Likert-type score.
- By nasal endoscopy.
- Other.
- I do not evaluate it.

2.3. Facial pain severity.

- I interview the patient about facial pain.
- Using a visual analogue scale (VAS) for facial pain.
- Using a Likert-type score.
- Other.
- I do not evaluate it.

2.4. Smell impairment severity.

- I interview the patient about smell impairment.
- Using a visual analogue scale (VAS) for smell impairment.
- Using a Likert-type score.
- Using validated smell tests (UPSIT, BAST, CCCRC, Sniffin' sticks).
- I do not evaluate it.
- Other.

2.5. The impact of CRSwNP on quality of life.

- I interview the patient about the impact of CRSwNP on different aspects of their daily life.
- Using a specific validated questionnaire: SNOT-22.
- Using a validated generic questionnaire: SF-36, EuroQoL-5 (EQ-5D).
- Other.
- I do not evaluate it.

2.6. Nasal polyp size.

- Using a 3-point Lildholdt score.
- Using a 4-point Nasal Polyp Score (NPS).
- Using the Lund-Kennedy or modified Lund-Kennedy score.
- Other
- I do not evaluate it.

1. What is the proportion of “severe uncontrolled” patients from the total of patients with CRSwNP that you currently visit?

- Less than 30%.
- 30–50%.
- 50–70%.
- More than 70%.

1. Which of the following tests do you currently carry out to define the inflammatory profile in a patient with CRSwNP? [select all that apply]

- Blood eosinophil count.
- Tissue eosinophil count (e.g. nasal cytology/nasal polyp biopsy).
- Determination of total IgE in blood.
- Presence of airway comorbidities.
- Smell impairment.
- Presence of allergic sensitization.
- Other.
- None.

1. What is the blood eosinophil count threshold that defines a patient with eosinophilic CRSwNP?

- > 150 eosinophils/µL.
- > 250 eosinophils/µL.
- > 300 eosinophils/µL.
- > 500 eosinophils/µL.

CRSwNP medical and surgical management

1. How do you evaluate CRSwNP control? [select all that apply]

- Using the European Position Paper on Rhinosinusitis and Nasal Polyps (EPOS) 2020 control criteria.
- Using the definition proposed in the Spanish Guide on Nasal Polyposis (POLINA) from 2011.
- Using the Sinusitis Control Test (SCT).
- Using an overall severity visual analogue scale (VAS).
- I interview the patient, without systematically following a score or test.
- Other.
- I do not evaluate it.

1. How long do you wait to assess the efficacy of a prescribed treatment?

I assess treatments every . weeks

1. What is your approach when a CRSwNP patient and shows lack of disease control with an adequate medical treatment in a follow-up appointment? [select all that apply]

- I confirm the diagnosis.
- I propose a surgical intervention.
- I modify the prescribed topical corticosteroid treatment regimen.
- I prescribe a cycle of oral/systemic corticosteroids.
- I consider prescribing a biological treatment.

1. What would be the maximum annual courses of oral corticosteroids that you would prescribe to a patient with CRSwNP before considering other options (e.g. surgery or prescription of a biological treatment)?

1 course/year. 2 courses/year. 3 courses/year. 4 or more courses/year.

1. What kind of primary surgery for CRSwNP do you perform more often?

- Polypectomy.
- Functional endoscopic sinus surgery (FESS).
- Extended endoscopic sinus surgery (EESS), involving frontal sinuses.
- Radical endoscopic sinus surgery (RESS) plus Draf III.

1. Do you use phenotype-based criteria to select the optimal surgical approach for a CRSwNP patient? [select all that apply]

- CRSwNP with non-type 2 inflammation (non-eosinophilic, without asthma or allergy) => polypectomy.
- CRSwNP with non-type 2 inflammation (non-eosinophilic, without asthma or allergy) => functional endoscopic sinus surgery.
- CRSwNP with moderate type 2 inflammation (eosinophilic) => functional endoscopic sinus surgery.
- CRSwNP with moderate type 2 inflammation (eosinophilic) => extended endoscopic sinus surgery (involving frontal sinuses).
- CRSwNP with severe type 2 inflammation (refractory eosinophilic, NSAID-exacerbated respiratory disease [N-ERD]) => radical endoscopic sinus surgery, reboot, nasalization, DRAF III.
- I do not agree with the phenotype-based approach.

1. After failing to achieve control using the adequate medical treatment, when would you consider prescribing a biological therapy in a patient with severe CRSwNP? [select all that apply]

- As an alternative to a primary surgery.
- In patients with a history of previous surgery and candidate for revision surgery.
- In patients classified with a poor prognosis (asthma and/or concomitant N-ERD) right after a primary surgery.
- Currently I do not consider the possibility of biological treatment for CRSwNP patients.

Follow-up

1. How often do you schedule routine follow-up appointments for patients with severe CRSwNP? [select all that apply]

Every month. Every 3–4 months. Every 6 months. Annually.

1. During a follow-up visit, do you usually ask the patient…?

- 14.1. …if they have self-medicated with oral corticosteroids for the management of CRSwNP since the last visit.


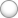
 Yes
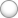
 No

- 14.2 …if they have been prescribed oral corticosteroids for the management of other conditions (e.g. asthma) since the last visit.


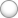
 Yes
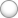
 No

1. What proportion of your CRSwNP patients are receiving maintenance treatment with oral corticosteroids for this or another condition?

- I do not visit any patients with this profile.
- Less than 25%.
- Between 25–50%.
- More than 50%.

1. How do you follow-up CRSwNP patients after a surgical intervention?

- I do not establish any particular follow-up. I prescribe daily intranasal corticosteroids and saline irrigations and discharge the patient.
- I prescribe daily intranasal corticosteroids and saline irrigations and refer the patient to Primary Care.
- I prescribe daily intranasal corticosteroids and saline irrigations and schedule an outpatient follow-up appointment.
- I follow a different process to those described above.
